# *Helicobacter pylori* Gastritis in Children—The Link between Endoscopy and Histology

**DOI:** 10.3390/jcm9030784

**Published:** 2020-03-13

**Authors:** Ana-Maria Teodora Domșa, Raluca Lupușoru, Dan Gheban, Radu Șerban, Cristina Maria Borzan

**Affiliations:** 1 Department of Pathology, “Iuliu Hațieganu” University of Medicine and Pharmacy, 400000 Cluj-Napoca, Romaniadgheban@gmail.com (D.G.); 2 Department of Gastroenterology and Hepatology, “Victor Babes” University of Medicine and Pharmacy, 300041 Timisoara, Romania; 3 Department of Functional Sciences, “Victor Babes” University of Medicine and Pharmacy, 300041 Timisoara, Romania; 4 Emergency Clinical Hospital for Children, 400000 Cluj-Napoca, Romania; radusorinserban35@gmail.com; 5 Department of Pediatrics, “Iuliu Hațieganu” University of Medicine and Pharmacy, 400000 Cluj-Napoca, Romania; 6 Department of Public Health and Management, “Iuliu Hațieganu” University of Medicine and Pharmacy, 400000 Cluj-Napoca, Romania; borzancristina@yahoo.com

**Keywords:** Helicobacter pylori, gastritis, children, endoscopy, histopathology

## Abstract

Background: The management of *Helicobacter pylori* (*H. pylori*) infection raises important challenges, still being the most common chronic infection worldwide in all age groups. In high-prevalence regions, paediatric patients need a specific focus, as the acquisition of the infection takes place in childhood. The objective of this study was to analyze the endoscopic and histopathologic changes of the gastric mucosa in *H. pylori* infected children. Material and Methods: A retrospective study was performed on consecutive paediatric patients, ranging from 0 to 18 years of age, who underwent an upper gastrointestinal endoscopy (UGE) for a period of 5 years, regardless of their symptomatology. Endoscopy reports and histological slides were reviewed and clinical, endoscopic, and histologic data were recorded. Results: A total of 248 patients were included in the study, 82 (33.06%) of them being *H. pylori* infected. There was no difference in age and symptoms between the infected and noninfected group. A significant association was found between the *H. pylori* infection and histopathological parameters such as acute and chronic inflammatory infiltrate. The bacterial load influences the intensity of inflammation (*p* < 0.001). The chronic inflammation was predominant, only 23.2% of the patients displayed acute inflammation (*p* < 0.0001). The topographic distribution of inflammation was dominated by pangastritis (*p* = 0.04) with 58.6% of the patients presenting similar degrees of inflammation both in the antrum and corpus. Conclusion: Endoscopic features such as nodularity of the antral mucosa (*p* < 0.05) along with histological findings as lymphoid follicles (*p* < 0.05) are suggestive of *H. pylori* infection. However, the concordance between the endoscopic and histological diagnosis is still far from perfect (Cohen’s k coefficient = 0.42), maintaining the need for an invasive approach in children.

## 1. Introduction

*Helicobacter pylori* (*H. pylori*) infection is a major public health problem, being the most frequent chronic infection in the world, both in adults and in children [1]. A dynamic epidemiology, with broad variations among regions of the globe is reported in recent years [2]. The Gram-negative rod, first isolated by Marshall and Warren in 1982 [3], is classified by the International Agency for Research on Cancer as a class 1 carcinogen [4]. In Romania, a developing country, it is estimated that about 65% of the adult population is infected, with a prevalence of around 40% in children [5,6,7] but few epidemiological data are available.

The chronic inflammatory changes and ultrastructural alterations of the gastric mucosa induced by the bacteria create a predisposition to cancer [8,9]. Intestinal metaplasia is considered the point of no return in the gastric carcinogenesis [10]. Therefore, the eradication of *H. pylori* should be obtained prior to developing advanced preneoplastic lesions in order to prevent gastric cancer from occurring [11]. 

Given that in poorly developed and developing countries, the population is infected during childhood, with a maximum incidence in the first 5 years of life [12] and that the age of acquisition influences the later adverse outcomes [13,14], premalignant lesions may start earlier in life with the onset of gastric cancer at young ages. Hence, objective data is needed to optimally time the medical intervention. Unlike adults, where symptomatology is somehow more specific, most infected children are asymptomatic or exhibit non-specific symptoms [15].

The current guidelines for the management of *H. pylori* infection in children recommend the diagnosis to be based on upper gastrointestinal endoscopy (UGE) with biopsies [16]. The updated Sydney Classification for gastritis [17] should be used for the assessment of the histopathological changes of the gastric mucosa [16]. 

The aim of our study was to evaluate the histopathological lesions and the association with the endoscopic alterations encountered in *H. pylori* infected children.

## 2. Materials and Methods

In this retrospective study, we reviewed consecutive cases of children (age 0–18 years) who underwent UGE at the 1st Paediatric Clinic, part of the Emergency Clinical University Hospital for Children in Cluj-Napoca, for a period of 5 years, between 01.01.2013 and 31.12.2017. No selective criteria were applied for the patients’ symptoms. The exclusion criteria were the age (patients above 18 years old were excluded) and medical history of *H. pylori* infection. After applying the exclusion criteria, 7 patients were removed from analysis and a total of 248 children and adolescents were considered eligible for the study.

The study was conducted according to the declaration of Helsinki, and the protocol was approved by the ethical committee of the University of Medicine and Pharmacy Cluj-Napoca (208/16.05.2017). Clinical, anthropometric, endoscopic and histological data were anonymously collected.

### 2.1. Endoscopy

Informed consent was obtained from the patients’ parents or legal guardians prior to the procedure. A single examiner performed the UGE on all 248 children, according to standard techniques. The purpose of the UGE was to investigate the patients’ digestive symptoms. The following macroscopic aspects of the stomach and duodenum were noted from the endoscopic records: normal appearance, hyperemia, edema, erosions, nodular and paving stone aspect, and reflux. For each patient, 3 biopsy specimens were obtained from the antrum, the body of the stomach, and the duodenum.

### 2.2. Histopathological Examination

All the available slides, stained with Haematoxilin and Eosin as well as Giemsa for the assessment of *H. pylori* status were reviewed by the same pathologist, who was unaware of the endoscopic findings. The patient was considered infected if Haematoxilin and Eosin along with the Giemsa stain were both positive. The samples were scored according to the updated Sydney System, and the following items were recorded: chronic inflammation, activity, atrophy, *H. pylori* colonization, and the presence of metaplasia.

Additionally, lymphoid follicles and erosions were noted. Pangastritis was diagnosed when inflammation in the antrum and in the corpus reached the same grade.

### 2.3. Statistical Analysis

Continuous data with normal distribution was presented as mean ± SD, continuous data without normal distribution was presented as median and interquartile range (IQR) and for nominal variables data was presented as percentages. The normality was tested using the Kolmogorov–Smirnov test. The differences between the groups were assessed using the student t-test for continuous variables with normal distribution, and the Mann–Whitney U test for continuous variables without normal distribution. The Fisher test and Pearson chi-squared test were used for proportions. The influence of different dichotomous variables was tested using univariate and multivariate logistic regression models. The comparison between two medical methods was assessed using the Spearman correlation coefficient and the Cohen inter-rater agreement (kappa). The performance of the methods was tested using receiver operating characteristics analysis (AUROC). For the statistical analysis we used SPSS v.17 (SPSS Inc., Chicago, IL, USA) and Microsoft Office 2019. A *p*-value < 0.05 was considered significant, at a 95% confidence level for intervals. 

## 3. Results

Of the 248 patients analyzed, 84 were male and 164 were female. The baseline patients’ characteristics are presented in Table 1. 

We observed that among the 248 patients, 82 (33.06%) exhibited *H. pylori* infection with the mean age (13.51 ± 2.79 years) being similar to the 166 (66.94%) patients without *H. pylori* infection (13.12 ± 3.32 years, *p* =0.36).

In the *H. pylori* positive group, 24/82 (30.2%) were male and 58/82 (69.8%) were female while in the *H. pylori* negative group 60/166 (36.1%) were male and 106/166 (63.9%) were female, without any statistical differences in gender, p=0.43.

We divided the cohort according to age group, such as early childhood (0–6 years), middle childhood (7–12 years), and adolescence (13–18 years). The detection of *H. pylori* infection was 0%, 15.6%, and 33.7%, respectively. The prevalence of *H. pylori* increased gradually with age, and it was statistically significant (*p* < 0.05). In addition, we observed that if we split the patients according to age group, gender and *H. pylori*, there were differences between the gender in groups 7–12 years and 13–18 years (Table 2). 

There were no differences in the complaints that led to endoscopy between the groups with *H. pylori* and without *H. pylori* (*p* > 0.05), with more than half of the patients having dyspeptic symptoms (Table 3). 

The endoscopic examination revealed a notable nodularity of the mucosa, paving stone aspect and edema in the *H. pylori* positive group (Table 4), while histopathology showed increased activity and chronic inflammation in the *H. pylori* positive group (Table 5).

We found 143 discordant cases regarding endoscopic and histologic findings, but the Cohen’s k coefficient calculated between the two methods (endoscopy and histopathology) to diagnose *H. pylori* infection, was 0.42, indicating a good agreement between them (Table 6).

**Figure 1:**
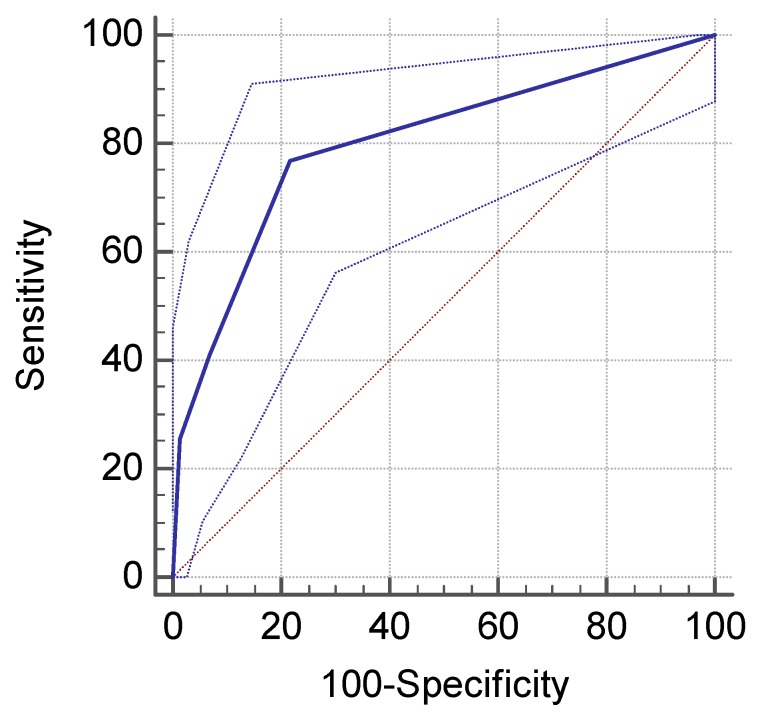
Performance of endoscopy in *H. pylori* infection detection.

Taking the histopathological exam as a “gold standard”, the endoscopic method for assessing *H. pylori* infection had a performance of AUROC = 0.80 (Figure 1), 95% CI (0.74–0.85), *p* = 0.0001, sensitivity = 76.9%, specificity = 78.3%, NPV = 94.3%, PPV = 42.3%.

Spearman’s correlation coefficient between endoscopic findings and histopathological findings was r = 0.43, *p* < 0.0001, indicating a direct moderately strong correlation. 

The nodular aspect observed at endoscopy was associated with the presence of *H. pylori*, regardless the colonization grade (*p* < 0.05). This aspect was also associated with moderate and severe activity at the histopathologic exam (*p* < 0.0001 and *p* = 0.001, respectively), and with moderate and severe chronic inflammation of the mucosa (*p* = 0.0004 and *p* < 0.0001, respectively). A severe activity increases the chance of developing this endoscopic aspect 17 times, OR = 17.33, 95% CI 1.75–171.28 and a moderate activity can raise the chance six times, OR = 6.20, 95% CI 2.42–15.9.

The paving stone aspect at endoscopy was associated with the detection of *H. pylori* (*p* = 0.03) and with the presence of follicles at the histopathologic exam (*p* = 0.04). In univariate analysis, the age group 0–6 years had three times the risk of developing a paving stone aspect of the mucosa than the rest of the groups (OR = 3.94, 95% CI 0.39–39.81). 

The congestive aspect and the presence of edema at endoscopy also increases with the *H. pylori* colonization grade (all *p* < 0.05).

Out of the 82 infected patients, 48 (58.6%) had *H. pylori* positive pangastritis, while 34 patients (41.4%) had *H. pylori* positive antral gastritis, *p* = 0.04. 

None of the endoscopic and histopathologic findings were associated with the diagnosis of antral predominant gastritis (*p* > 0.05). Conversely, pangastritis was associated with the activity, regardless of grade (*p* < 0.0001), with the chronic inflammation (*p* < 0.0001), with the presence of *H. pylori*, no matter the density (*p* < 0.0001), and with a nodular pattern at endoscopy (*p* < 0.0001), edema (*p* = 0.03) and congestion (*p* < 0.0001). Furthermore, pangastritis was associated with older ages, 30 (62.5%) of the *H. pylori* positive patients in the age group 13–18 years presented this pattern, *p* = 0.02.

Regarding the inflammatory pattern, 63 (76.8%) of the patients presented only chronic inflammatory infiltrate, while 19 (23.2%) also displayed acute inflammation (*p* < 0.0001). 

The severity of the chronic inflammation increased with the *H. pylori* colonization grade (*p* < 0.001). Mild chronic inflammation was associated with the presence of *H. pylori* infection only in the age group 7–12 years (*p* = 0.01). Moderate and severe chronic inflammation as well as mild and moderate activity were associated with the presence of *H. pylori* in the groups 7–12 and 13–18 years, *p* < 0.0001. Moderate chronic inflammation in the group 7–12 years was associated with a mild and moderate bacterial load (*p* < 0.0001), while in the 13–18 years group with all *H. pylori* densities, *p* < 0.0001.

The activity was associated with the presence of *H. pylori* regardless of the colonization grade. The activity severity also increased with the *H. pylori* density (*p* < 0.0001).

Lymphoid follicles were noted in 40 cases. Out of these patients, 11 (27.5%) had a high bacterial load (*p* = 0.001). Comparing the presence of lymphoid follicles with the degree of chronic inflammation, six (15%) of the patients had mild inflammation, 14 (35%) had moderate inflammation and 17 (42.5%) had severe inflammation, *p* = 0.01.

Among the 20 cases with microscopical gastric erosions, seven (35%) of them had *H. pylori* infection, *p* < 0.0001.

We found 36 cases of atrophy, 25 of them being graded as mild atrophy; nine (25%) of these patients were *H. pylori* positive (*p* < 0.0001). The presence of gastric mucosal atrophy increases with age (*p* < 0.01). Intestinal metaplasia was observed in three cases, with no relation to the *H. pylori* infection (*p* = 0.99).

## 4. Discussion

In this study we assessed the association between the *H. pylori* infection in children and the endoscopic and histopathological patterns determined by the presence of the bacteria.

Approximately one third of the analyzed patients had the *H. pylori* infection, with no significant mean age difference (*p* = 0.36) between the infected and the noninfected groups, even though previous studies state older age as a risk factor for the infection [18]. However, we also noticed the characteristic increase in the frequency of infection along with age (*p* < 0.05).

Our results confirm the varied spectrum of symptoms in children infected with *H. pylori* [19], with no significant difference being observed between the infected and the noninfected groups. 

The vast majority of studies report a fair agreement between the endoscopic and histological diagnosis of gastritis, particularly in children and support the routine collection of biopsy samples during endoscopy in pediatric patients [20,21,22]. This high discrepancy might stem from the differences in the geographic distribution of *H. pylori* infection and not necessarily from the endoscopist’s experience [23]. In contrast to other studies, the Cohen’s k coefficient of 0.42 determined in our analysis indicates a good agreement between the two methods; this could be explained by the high prevalence of infection in our country.

With respect to the nodular aspect of the antral mucosa in infected patients, there is a consensus in the literature stating that antral nodularity is a predictor of the *H. pylori* infection in children [24,25,26]. Our results confirm that antral nodularity observed at endoscopy is linked to the presence of *H. pylori* (*p* < 0.05), and also predict a higher activity grade and moderate to severe chronic inflammation of the gastric mucosa.

The histopathological parameters significantly associated with *H. pylori* infection were chronic and acute inflammation (*p* < 0.0001). A high bacterial load increases the intensity of the chronic (*p* < 0.001) as well as of the acute inflammatory infiltrate (*p* < 0.0001). When assessing the topographic distribution of the inflammation, we noticed a significant predominance of pangastritis (*p* = 0.04). Our observation contrasts with most of the previous reports that point out that histopathological changes are primarily limited to the antral mucosa [27].

When analyzing the inflammatory pattern, since the first published studies, it has been demonstrated that when compared to adults, the lymphocytic component is predominant in the inflammatory response of children; they usually present a paucity of the acute inflammation and a high colonization grade [28,29,30,31,32]. These data are supported by the results of our study with only 23.2% of the patients presenting activity (*p* < 0.0001). This is the reason why caution is recommended when extrapolating histopathological aspects encountered in adults to children [33].

Another important observation made by most of the researchers, and confirmed by our study, is that the occurrence of lymphoid follicles in the gastric mucosa is indicative of *H. pylori* infection and their presence is significantly associated with the density of *H. pylori* and with the grades of the acute and chronic inflammatory response [24,34].

In areas with a high prevalence of infection and high rates of gastric cancer, children have greater lesions and higher *H. pylori* density rates [35]. Apparently, the rarity of gastric ulcers related to the infection is due to the particularities of the inflammatory response [36]. Previous investigators agree on the association between duodenal ulcers and erosions and *H. pylori* infection; no link could be established between gastric ulcers and the infection [18,37,38]. As supported by our analysis, a relationship can be established between gastric erosions, not ulcers, and *H. pylori* infection in children [18].

Among studies examining atrophy and intestinal metaplasia in children, the reported rates vary greatly and the association of these changes with the infection is controversial [39]. Our data suggest that there is a link between atrophy and *H. pylori* infection. However, we should take into consideration the limited number of cases, the fact that atrophy was graded as mild in 69.44% of the cases and the great variability between investigators when reporting mild atrophy [40].

From the eight suspected cases of celiac disease and two suspected cases of Crohn disease, three and one of the cases were *H. pylori*-positive, respectively. As described in previous studies, especially in high-prevalence areas, the infection can be an incidental finding during endoscopy performed for the diagnosis of celiac disease or inflammatory bowel disease [41].

We admit that our study has some limitations. Since it was conducted in a retrospective manner, we had incomplete data regarding patients’ associated diseases, medication, and previous *H. pylori* eradication therapy. Because of the fact that this study is hospital-based and patients who underwent endoscopy had various gastrointestinal symptoms, and knowing that the infection is asymptomatic in the vast majority of cases [42], prevalence data cannot be extrapolated to the general pediatric population.

The current guidelines recommend a diagnosis based on culture or histopathology along with one other positive biopsy-based test and to obtain at least six gastric biopsies [16]. So, our detection methods could be associated with an underdiagnosis of the infection.

This was a single-center study, with a limited number of cases and short time span. Given the fact that Romania is considered to have a high prevalence of the infection [2] and a high risk of gastric cancer [43] and considering that limited research has been published on this topic and that dated epidemiology is available [44], it is advisable for researchers in our country to conduct multicenter, standardized, nationally representative surveys.

## 5. Conclusions

Our data show a good agreement between endoscopy and histology when evaluating *H. pylori* gastritis in children, but a high rate of discordance between the two methods still remains, as *H. pylori* can be seen in a gastric mucosa with a normal endoscopic appearance.

The endoscopic nodular aspect of the antral mucosa highly predicts the infection and higher grades of inflammation in the mucosa. In addition, the identification of lymphoid follicles in histology is linked to the presence of *H. pylori*, both qualitative and quantitative and also to the grades of acute and chronic inflammation. We also found that pangastritis is the predominant topographic pattern in infected children in our study.

The present study reinforces the fact that invasive methods, such as endoscopy with biopsy, should remain the “criterion standard” for diagnosing *H. pylori* infection in children. Diagnostic accuracy can be increased by adhering to the current international recommendations.

Considering the high rates of gastric cancer in developing countries and knowing that the disease outcome is dependent on the age at which the bacteria is acquired, we are in urgent need of prospective national studies to evaluate the prevalence of the infection and the antibiotic resistance profiles, in order to be able to provide individualized detection, tailored therapy and to identify the children who need closer surveillance.

## Figures and Tables

**Table 1:** Patients’ characteristics.

Variable	Median (Range), n (%)
Gender	
Male	84 (33.8%)
Female	164 (66.2%)
Age (years)	14 (0-18)
0–6	4 (1.6%)
7–12	94 (37.9%)
13–18	150 (60.5%)

**Table 2:** Comparison between the *H. pylori* positive group and *H. pylori* negative group according to age groups.

Age Group	*Helicobacter Pylori* Positive (*H. pylori*+)	*Helicobacter Pylori* Negative (*H. pylori*-)	*p*-value
0–6			
Male	0	3 (75%)	0.14
Female	0	1 (25%)	0.99
7–12			
Female	14 (14.8%)	40 (42.5%)	0.0001
Male	12 (12.7%)	28 (30%)	0.006
13–18			
Female	44 (29.5%)	65 (43.3%)	0.01
Male	12 (8%)	29 (19.2%)	0.007

**Table 3:** Reasons for endoscopy in children with or without *H. pylori* infection.

Symptoms	*H. pylori*- *n* = 166	*H. pylori*+ *n* = 82	*p*-value
Dyspeptic syndrome	102 (62%)	52 (63.4%)	0.90
Abdominal pain	28 (17.4%)	22 (26.8%)	0.1
Emesis	9 (5.4%)	4 (4.8%)	0.91
Anemia	3 (1.8%)	5 (6%)	0.16
Suspected celiac disease	5 (3%)	3 (3.6%)	0.89
Catabolic syndrome	9 (5.4%)	2 (2.4%)	0.45
Upper digestive hemorrhage	0	2 (2.4%)	0.21
Gastro-esophageal reflux	3 (1.8%)	0	0.54
Suspected Crohn disease	1 (0.6%)	1 (1.2%)	0.79
Anorexia	1 (0.6%)	1 (1.2%)	0.79

**Table 4:** Endoscopic pattern of the gastric mucosa in children with or without *H. pylori* infection.

Endoscopic Pattern	*H. pylori*- *n* = 166	*H. pylori*+ *n* = 82	*p*-value
Minimal changes	148 (89.1%)	41 (50%)	<0.0001
Nodularity	9 (5.4%)	30 (36.5%)	<0.0001
Paving stone	9 (5.4%)	11 (13.4%)	0.04
Hyperemia	163 (98.1%)	81 (98.7%)	0.85
Edema	76 (45.7%)	50 (60.9%)	0.03
Erosions	11 (6.6%)	4 (4.8%)	0.78

**Table 5:** Histopathological findings in the gastric mucosa in children with and without *H. pylori* infection.

Histopathological Findings	*H. pylori*- *n* = 166	*H. pylori*+ *n* = 82	*p*-value
Activity	6 (3.7%)	82 (100%)	<0.001
Chronic inflammation	50 (31.4%)	50 (60.9%)	<0.0001
Atrophy	27 (75%)	9 (25%)	<0.0001
Intestinal metaplasia	1 (0.6%)	2 (2.4%)	0.54

**Table 6:** Comparative analysis between endoscopic and histopathologic findings.

Endoscopic Findings	Histopathologic Findings	No. Biopsies	*H. Pylori*+
Minimal, non-characteristic changes	Normal	46 (24.3%)	0
	Abnormal	143 (75.7%)	40
	Chronic inactive gastritis	87	16
	Chronic active gastritis	29	24
	Other	27	0
